# Philosophy, Ethics, and Humanities in Medicine: Expanding the open-access conversation on health care

**DOI:** 10.1186/1747-5341-1-1

**Published:** 2006-03-17

**Authors:** Dan J Stein, Derek Bolton, Damiaan Denys, Thomas Huddle, Tia Powell

**Affiliations:** 1 Dept of Psychiatry, University of Cape Town, Groote Schuur Hospital J-2, Anzio Rd, Observatory 7925, Cape Town, South Africa; 2 Dept of Psychology, Institute of Psychiatry, Kings College London, London, UK; 3 Dept of Psychiatry, University Medical Center, Utrecht, Netherlands; 4 Division of General Internal Medicine, University of Alabama at Birmingham, Birmingham AL USA; 5 New York State Task Force on Life and the Law, New York, USA

## Abstract

Natural philosophy once spanned the fields of philosophy, science, and medicine. Scientific disciplines and medical specialties have rapidly achieved independence, and the availability of the internet and open-access publishing promises a further expansion of knowledge. Nevertheless, a consideration of the grounding concepts and ethical principles that underlie health care remains paramount. It is timely, therefore, to contribute to the global conversation on health care with an open-access journal that focuses on addressing the conceptual basis of medicine and related disciplines, considering the ethical aspects of clinical practice, and exploring its intersection with the humanities (including history of medicine).

## 

Open-access publishing has been a spectacular innovation for science and medicine, giving readers rapid and free access to new information, and launching a global conversation on health care. Health care researchers and practitioners have, in turn, busily produced new basic experiments, clinical trials, and reviews and guidelines for publication on the world-wide web, spurring further virtual debate and discussion. It is timely, we believe, to contribute to this conversation by making space for an open-access journal that takes a particularly broad view of health care, addressing the conceptual basis of medicine and related disciplines, considering the ethical aspects of clinical practice, and exploring its intersection with the humanities (including history of medicine).

Those interested in the conceptual and historical roots of medicine will not need reminding that early on work in natural philosophy spanned philosophy, science, and medicine. While scientific advances have led to the development of an ever-growing range of scientific disciplines and medical specialties, a consideration of the grounding concepts and ethical principles that underlie health care remains as crucial as ever. Indeed, advances in knowledge, and changes in practice, mean that these grounding concepts and ethical principles require constant reconsideration and reworking. This, then, is the work of philosophy of medicine, of bioethics, and of work at the overlap between the clinic and the humanities.

To our knowledge *Philosophy, Ethics, and Humanities in Medicine *is the first open-access journal that aims to expand the discussion on health care by focusing on the intersection between philosophy, ethics, and the humanities and clinical theory and practice. The arguments for open access have been outlined previously; authors are ensured of wide dissemination of their work and are at liberty to further reproduce and disseminate their articles, readers can find articles on standard search engines and obtain ready access regardless of the quality of their libraries, and taxpayers are easily able to review the outputs of publicly funded research [1]. The advantages of open access are particularly apparent in the developing world, where libraries are few and budgets are low.

In the spirit of open-access publishing and the internet conversation on health care, we will encourage a broad range of submissions, hoping to attract authors from different disciplines. Details of our editorial board and editorial policy are available on the journal's website [2]. The journal will follow the BioMed Central Open Access Charter [3]; articles accepted for publication are immediately accessible, authors hold the copyright and grant the right to reproduce and disseminate work, and a copy of the full-text of each article is immediately archived in the US National Library of Medicine's full-text repository [4], as well as in repositories at the University of Potsdam in Germany [5], at INIST in France [6], and in e-Depot, the National Library of the Netherlands' digital archive of all electronic publications [7].

We hope that those working at the intersection between philosophy, ethics, the humanities and clinical theory and practice will become increasingly aware of the value of open-access publication, and will use the journal to contribute to the ongoing global conversation on health care. We are aware, on the other hand, that this is an intersection that has relatively little funding and few full-time researchers, and so attracting a steady flow of articles and discussion represents a considerable challenge. But if the good practice of medicine necessarily requires a good conceptual base – then we have little choice but to launch this journal, and to attempt to extend the virtual debate on health care to address its foundational constructs and practices.

## Competing interests

Dan Stein has received research grants and/or consultancy honoraria from Astrazeneca, Eli-Lilly, GlaxoSmithKline, Lundbeck, Orion, Pfizer, Pharmacia, Roche, Servier, Solvay, Sumitomo, and Wyeth.
